# The Implantation of Teeth in Artificial Sockets

**Published:** 1888-11

**Authors:** G. W. Weld

**Affiliations:** New York.


ARTICLE III.

### THE IMPLANTATION OF TEETH IN ARTIFICIAL SOCKETS.

DR. G. W. WELD, NEW YORK.

It seems to me that all the advocates and enthusiasts of the implantation of teeth can hope for in the way of success is to be compared only with the success which, in the past has been associated with replanting and transplanting,

The experience of a number of our professional brethren who have experimented in this direction under favorable conditions has, in the large majority of cases, resulted only in failure.

Of course it may be argued that the operation is altogether new, and that transplanting is no criterion by which the success, or non-success, of *implanting* can be judged. To some extent this may be so. It has been said for M. Pasteur in his novel cure for hydrophobia that no one, without experience, could rightfully gainsay his propositions, till time had demonstrated either their truth or falsity. So far as this argument goes, both M. Pasteur and Dr. Younger have had an advantage over those who have taken exceptions to their methods.

It is possible, so far as Dr. Younger's Practice is concerned, that more time will be required to determine its worth.

Admitting that a tooth may be retained in a new socket made in the alveolus is only to acknowledge the sequel, or the succeeding part of transplantation.

Let us briefly analyze Dr. Younger's progress and present position on the subject.

First.—His experience dates from the 13th of June, 1885—a little less than two years, and about the same length of time that those who have experimented in transplanting begin to realize that the ultimate end of all their efforts is a failure.

Second.—He claims that he has had wonderful success; but every one who has transplanted has had and claimed, at first the very same thing.

Third.—The want of success in transplanted teeth has been caused by the absorption of their roots ; a condition of affairs which Dr. Younger, in one case, has admitted that he has observed on the roots of *implanted* teeth. Thus we find, in looking at the practice from a practical standpoint, that while the Doctor cheerfully recommends for adoption implantation, as being more permanent than transplantation, he, at the same time, "out of his own mouth," virtually places it on the same level.

With this understanding of the question it seems hardly necessary to touch on the *rationale* of the process of repair. Those who have experimented and made observations, and tabulated their results, know that the roots of either a replanted or transplanted tooth will, after some time, in a great majority of cases, become partially absorbed, and that this is the direct cause of their "dropping out." Dr. Waters, of Boston, who has experimented largely in this direction, found this condition in almost every case.

It does not seem reasonable to suppose that Dr. Younger's theory regarding the reattachment is well founded, or that it will ever be adopted by the profession. In fact he has already in a frank and good natured manner acknowledged that he "had to make a theory" to fit the case, and that the theory of revitalization of the pericementum suggested itself to him as being the most natural. It seems more probable that when an implanted tooth becomes reattached to the jaw bone it is only in virtue of its being tolerated as a foreign body precisely as other foreign bodies in other parts of the system have been known to be tolerated, and become incapsuled in the jaw, is unquestionably a fact; but this, by itself from a practical standpoint, proves nothing regarding its future, inasmuch as both replanted and transplanted teeth, as above stated, are similarly retained.

In an article recently published in the "*American System of Dentistry*," I have stated that the process by which the "erosions on the roots of replanted teeth are formed is not fully determined. Dr. Rollins states that 'the microscopical changes wrought by it cannot be distinguished from the absorption seen in deciduous teeth with living pulps,' and claims that 'these lunar excavations seen in teeth with *dead* pulps are produced, as they are admitted to be in teeth with *living* pulps, by the agency of living cells.'"

This is the teaching of modern pathology, the probability being that these cells, "giant cells," or "osteoclasts," secrete erosive fluids, which, acting on the organic as well as the inorganic constituents of the devitalized tooth substance, produce their gradual decomposition, solution and absorption. The process has its parallel in the erosion found on osseous sequestra which have been imbedded in living tissue.

The conclusions which I have arrived at with regard to the practice and phenomena of replantation (which hold good either for implantation or transplantation) may be thus summed up:

First—"That these teeth will, as a rule, reattach themselves, and become firm in the alveolar sockets from which they were taken; but the length of time they remain in a useful condition varies in each case, and is dependent on the constitution and age of the patient and local and systematic conditions.

Second—"That a full vital relation may sometimes, though rarely, be re-established; but that usually it exists only during that period of time after extraction when the root approximates a natural and normal condition, and terminates with the death of the peridental membrane and the protoplasmic constituents of the cement and dentine; the tooth then becoming a foreign body, and gradually undergoing disintegration through the action of giant-cells or osteoclasts.

Third—"That devitalization of the peridental membrane, cement and dentine usually speedily follows replantation, the tooth being then retained in position by fibrous connective tissue and the granulations from the soft tissues penetrating the lacune and canaliculi of the cement.

Fourth—"That the severance of this connection and the failure the operator may be caused either by the gradual loosening, resulting from continued oscilation, or to the supervention of acute inflammatory processes to be arrested only by the removal of the foreign body.

Fifth—" That in view of the possibility of danger and the certainty of ultimate failure attending this class of operations, the practice of replantation, either for the purpose of filling sensitive cavities, the treatment of neuralgia, or of alveolar abscess, or for the purpose of engrafting porcelain or other crowns, is unjustifiable."—*Items of Interest.*

---

ARTICLE IV.

### A FEW ITEMS OF IMPORTANCE.

